# Supplementary material for: Information advantage in sensing revealed by Fano-resonant Fourier scatterometry
Source: Nat Commun. 2025 Dec 10;16:11388. doi: 10.1038/s41467-025-66187-9 (PMC12738711; doi:10.1038/s41467-025-66187-9)
Supplement: Supplementary file 1 — Supplementary Information [file 41467_2025_66187_MOESM1_ESM.pdf]

Supplementary information for "Information advantage in sensing revealed by Fano resonant Fourier scatterometry"

Nick Feldman,† Arie J. den Boef,‡,¶,§ Lyubov V. Amitonova,‡,¶ and A. Femius Koenderink*,†

†*Department of Information in Matter and Center for Nanophotonics, AMOLF, Science Park 104, 1098 XG, Amsterdam, Netherlands*

‡*Metrology department, Advanced Research Center for Nanolithography (ARCNL), Science Park 106, 1098 XG Amsterdam, The Netherlands*

¶*Department of Physics and Astronomy, and LaserLaB, Vrije Universiteit, De Boelelaan 1081, 1081 HV Amsterdam, The Netherlands*

§*ASML Netherlands B.V., De Run 6501, 5504 DR, Veldhoven, The Netherlands*

E-mail: f.koenderink@amolf.nl

Supplementary note 1: Experimental setup

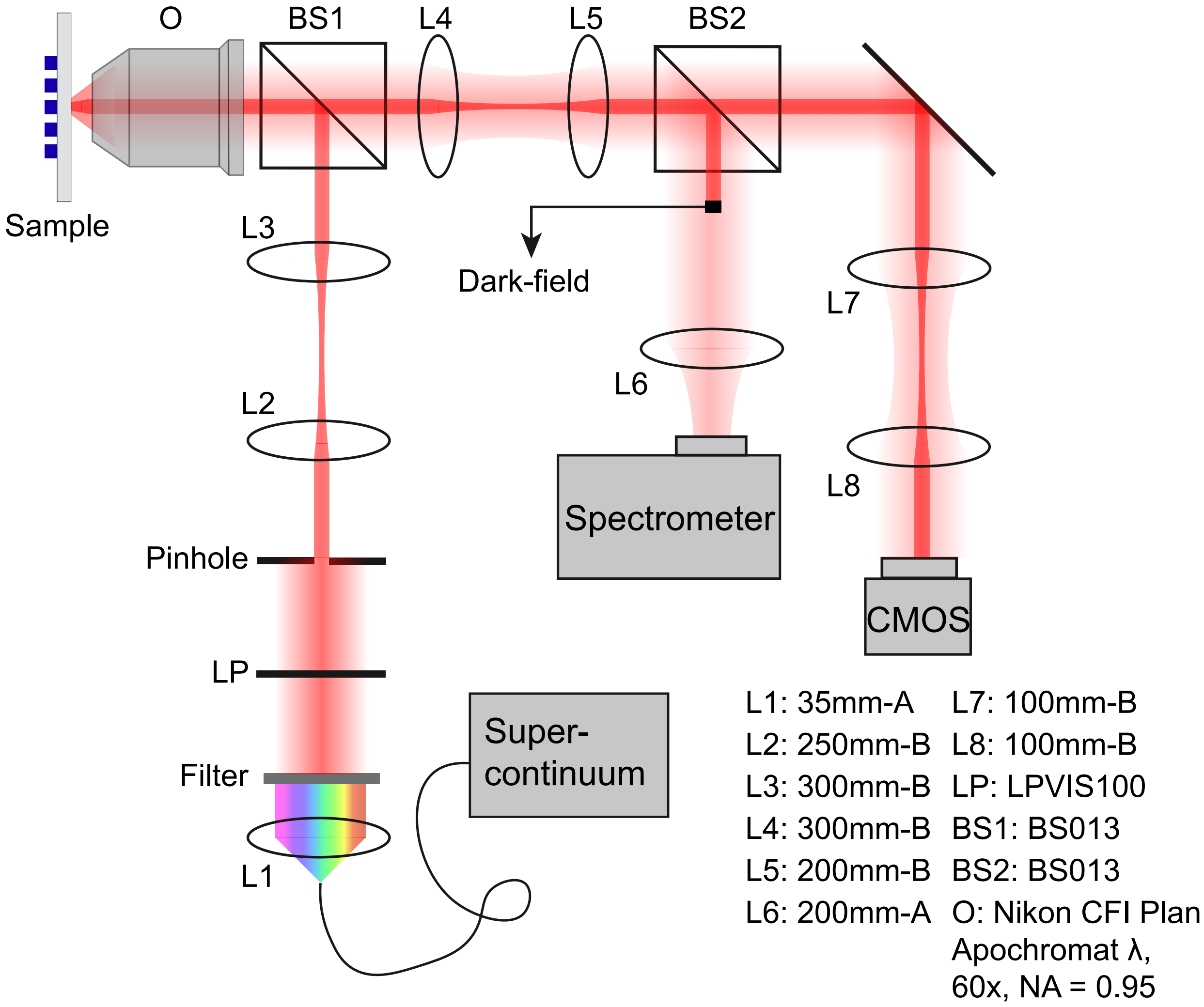

Supplementary Fig. 1: Experimental setup for dark-field spectroscopy and Fourier microscopy. Sketch of the complete experimental setup as used in the experiments from the main text. All lenses are achromatic (AC-254), and the indicated codes list the focal length in millimeters, followed by an A or B for the antireflection coating (Thorlabs nomenclature).

The experimental setup that is used to perform dark-field spectroscopy and Fourier scatterometry is depicted in Supplementary Fig. 1. A supercontinuum laser (NKT Whitelase Micro) is coupled into a single mode optical fiber and collimated into a Gaussian beam. The beam is subsequently linearly polarized and clipped in size by a 500 μm pinhole. The pinhole is relay imaged by a pair of lenses into the pupil plane of a high NA (NA $= 0.95$) microscope

objective, effectively reducing the NA of the illumination beam to approximately 0.18. The beam is then focused onto the sample plane such that a single metaring is entirely illuminated, after which the objective captures both the backreflection of the illumination and the scattered signal originating from the sample. In the dark-field spectroscopic measurement modality, the entire spectrum of the supercontinuum laser is illuminating the sample. Here, the direct reflection of the laser is filtered out by conjugating the pupil plane of the objective by a pair of lenses and placing a hard mask in this intermediate image plane, such that after the mask only scattered light is propagating through the system. This scattered signal is then focused onto the slit of a spectrometer (Andor Shamrock 303i) equipped with a cooled CCD (Andor iVac), after which a dark-field spectrum can be acquired. All spectra in the main text have been acquired with 10 ms integration time and summing 100 spectra. For the Fourier scatterometry measurements, the illumination beam is color filtered by bandpass filters with 10 nm bandwidth. The same scattered signal is captured by the microscope objective, where the pupil plane of the objective is now relay imaged onto a C-MOS camera (Basler, acA1920–40um). The direct reflection of the low NA illumination beam is now computationally clipped in a postprocessing step. In all the data shown in the main text, Fourier plane images are acquired at 500 ms integration time. All lenses, polarizers and beamsplitters shown in the figure have been bought from Thorlabs, and their respective serial numbers can be found next to the sketch of the setup. Sample navigation is controlled by Thorlabs motorized actuators (Z812) along with K-Cube steppercontrollers (KST101), and a 3D nanopositioning piezo (PI Nanocube) for fine alignment.

Supplementary note 2: Camera calibration and noise characterization

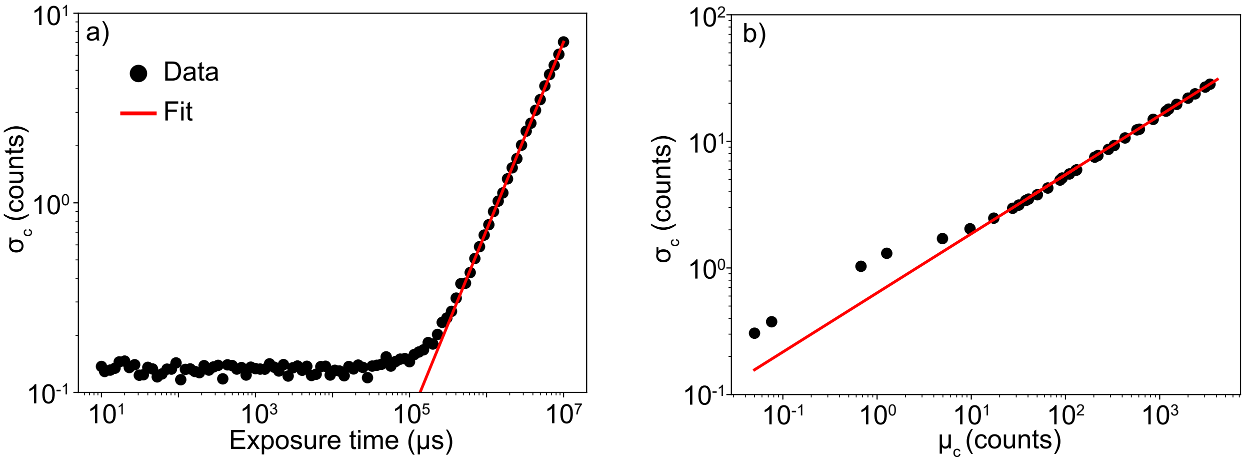

Supplementary Fig. 2: **Camera calibration and noise characterization.** Panel (A): Standard deviation of the counts in an image acquired in complete darkness versus exposure time. Panel (B): Standard deviation of the counts in an image versus the mean of the counts in the same image, acquired at a constant exposure time of 500ms. In every image, the illumination conditions are varied in intensity. The data in both panels are fitted by power laws.

To characterize the noise in our sensing experiment, we calibrated our image sensor based on a standard procedure.[1] First, we characterized the dark-noise of our camera by acquiring a series of images in complete darkness while varying the exposure time. From the resulting image we extract the noise by taking the standard deviation of the counts σ_c in the image, and plot the result of all these images versus the exposure time in Supplementary Figure 2a in a logarithmic plot. For high exposure times the noise follows a linear trend, which is verified by fitting a power law through the datapoints, resulting in the fitcurve $\sigma_c = (8.32 \times 10^{-7}) \cdot t^{0.99}$. This behavior is typical for high exposure times, where dark noise in the form of thermally induced photo-electrons are generated. For lower exposure times, the curve flattens towards a constant offset, which describes the read-out noise of our detector.

Next, we characterize the photon transfer curve of the detector at a constant exposure

time equal to 500 ms (equal to the exposure time of all acquired images in the main text). To achieve a constant illumination profile, we place a 1 cm block of Teflon in front of the camera which acts as a diffuser, and illuminate the Teflon block with a halogen lightsource. We then acquire several images for varying illumination intensities, which we control by linear polarizers. The photon transfer curve is retrieved by plotting the standard deviation of the counts σ_c in the acquired images versus the mean of the counts μ_c, which is shown in Supplementary Figure 2b in a logarithmic plot. From the curve, we van discern three regimes. The first regime, for mean counts values higher than approximately 10 counts, we observe a trend which can be fitted by a powerlaw of $\sigma_c = 0.63 \cdot \mu_c^{0.47}$. This trend is reminiscent of Poissonian statistics, as the standard deviation scales with the square root of the mean, and thereby describes the shot-noise limited regime of the experiment. From the fit, we can extract the gain of the image sensor, which is equal to 2.52 e^-/DU. For mean count values below circa 5 counts per pixel the trend is still Poissonian, but with a ca. 2-fold higher prefactor (same slope, but constant offset in logarithmic plot). We attribute this to an automatic switch in gain settings at low count rates specific for this camera. Finally, for even lower mean count values, the noise again reaches the constant offset defined by the readout noise.

Supplementary note 3: Different Fano structures and perturbation types

In this section, we theoretically investigate the potential information advantage of Fano resonant Fourier scatterometry beyond the specific meta ring and perturbation type described in the main text. To this end, we perform coupled dipole calculations on three well-known types of Fano-resonant structures, namely the dielectric meta ring described in the main text, a dielectric oligomer of nanospheres, and a plasmonic cluster of nanorods, as shown in Supplementary Figure 3. These structures are subsequently imbued by three different

types of perturbations that are frequently encountered in nanoscale sensing experiments, such as structural displacements of nanoparticles Δy in Supplementary Figures 3a-3c, a homogeneous refractive index perturbation Δn in Supplementary Figures 3d-3f, and a localized dielectric perturbation in the form of a single polarizable object $\Delta\epsilon$ in Supplementary Figures 3g-3i. The meta ring is modeled according to the modeling parameters discussed in the Methods section of the main manuscript, the dielectric oligomer is modeled by spherical particles of radius $R = 80$ nm with a dielectric permittivity of $\epsilon = 12$ and an interparticle gap size of 20 nm, and the plasmonic cluster is modeled by ellipsoidal particles with long axis $L = 130$ nm and short axis $W = 50$ nm. The permittivity of the plasmonic rods is described by a Drude model according to $\epsilon = \epsilon_\infty - \omega_p^2/(\omega^2 + i\omega\gamma)$, where $\epsilon_\infty = 1$, $\omega_p = 8.2 \times 10^{15}$ rad/s and $\gamma = 8.3 \times 10^{13}$ rad/s. All unperturbed structures are embedded in vacuum. For the interlaced displacements, the perturbation strength corresponds to $\Delta y = 10$ nm, where the perturbed structures are highlighted in red in Supplementary Figures 3a-3c.

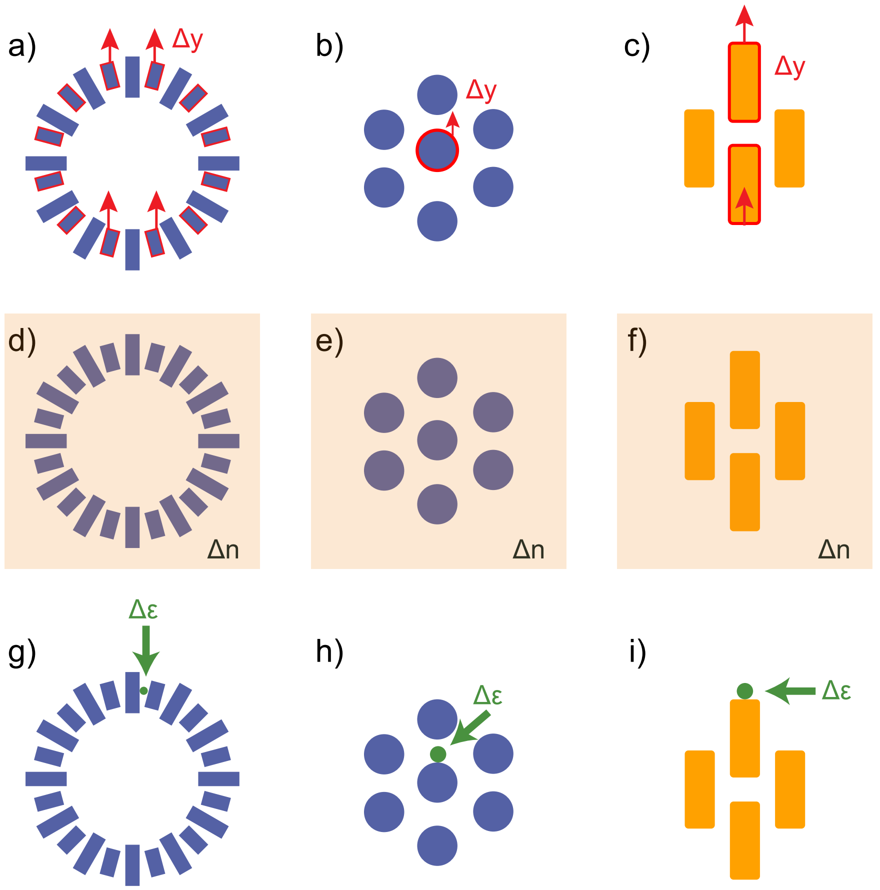

Supplementary Fig. 3: **Different Fano resonant structures and perturbation types.** Panels (A)-(C) denote dielectric metaring, dielectric oligomer and plasmonic cluster of nanorods respectively, where the perturbation corresponds to an upward shift Δy of individual meta atoms as indicated in the Figure. (D)-(F): Same as panels (A)-(C), but here the perturbation corresponds to a homogeneous refractive index perturbation. (G)-(I): Same as panels (A)-(C), but here the perturbation corresponds to a localized dielectric perturbation in the form of a single polarizable object.

The homogeneous refractive index perturbation corresponds to $\Delta n = 0.01$. Finally, the localized dielectric perturbation corresponds to a dielectric sphere of refractive index $n = 1.4$ and radius $R = 10$ nm, where the position of the nanosphere is indicated in Supplementary Figures 3g-3i. All structures are excited by a y-polarized plane wave at normal incidence, and the scattered signal is calculated in reflection.

Supplementary Figure 4 shows the relevant calculated observables in the form of scattering spectra in Supplementary Figures 4a-4c for the dielectric meta ring, the dielectric

oligomer and the plasmonic cluster respectively, where a Fano lineshape is present for all structures.

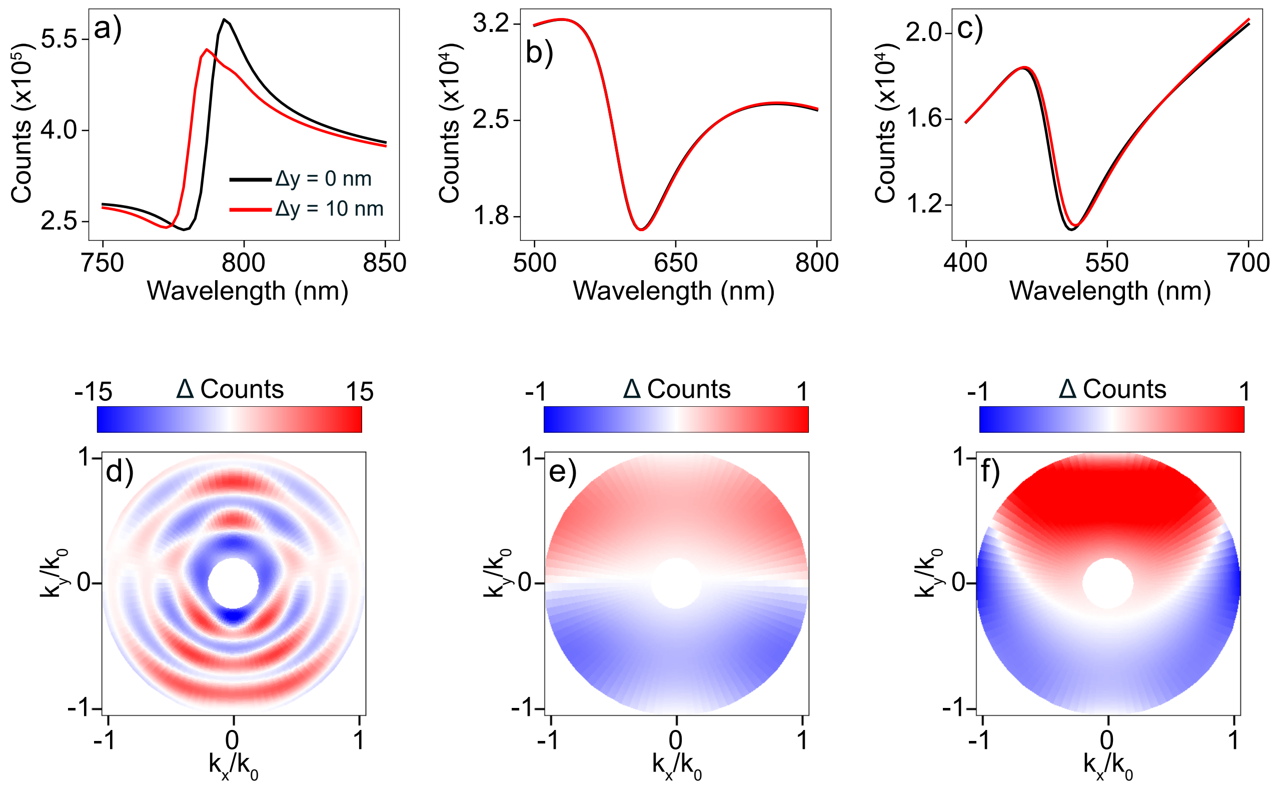

Supplementary Fig. 4: **Scattering spectroscopy and Fourier scatterometry.** Panels (A)-(C) denote scattering spectra for the dielectric metaring, the dielectric oligomer and the plasmonic cluster respectively. Black and red curves denote unperturbed and perturbed scattering spectra respectively, where the perturbation strength is set to $\Delta y = 10$ nm. Panels (D)-(F) denote differential Fourier space scatterometry images for the dielectric metaring, the dielectric oligomer and the plasmonic cluster respectively, where the wavelength is set to (D): 775 nm, (E): 640 nm, and (F): 500 nm.

Black and red curves denote unperturbed and perturbed spectra respectively, where the perturbation corresponds to the upwards shift of individual particles by $\Delta y = 10$ nm. We next calculate differential Fourier space images for the respective structures acquired at the Fano resonance, and show the results in Supplementary Figures 4d-4f, where the wavelengths are set to 775 nm, 640 nm and 500 nm respectively. Clear directional scattering effects can be appreciated for all three different Fano structures.

To finally quantify the information advantage these directional scattering effects have

compared to the angle integrated scattering spectra, we calculate the Fisher information of both a Fourier space image and a scattering spectrum according to the workflow of Figure 5 described in the main text. We assume that shot-noise is the only noise contribution in the calculation, such that the expression for the Fisher information becomes:

$$F = \frac{1}{I_0}\left(\frac{\partial I}{\partial \theta}\right)^2 \tag{1}$$

Supplementary Figure 5 shows the information comparison for all Fano-resonant structures and perturbation types. The red diamonds and blue circles indicate the Fisher information of an angle resolved and angle integrated calculation respectively, and the panel labels correspond to calculation results performed on geometries that conform to the labeling of Supplementary Figure 3. First of all, it can be seen that for all structures and perturbation types, the Fourier space readout is more informative that the angle integrated measurement. The information advantage of the dielectric meta ring in the case of a structural perturbation Δy as shown in Supplementary Figure 5a is 19-fold, providing the same order of magnitude information advantage as demonstrated experimentally in the main text, especially when the absence of fabrication noise in the calculation is taken into account. Finally, the information advantage of Fourier space readout seems to be most effective when the perturbation induces an asymmetry in the Fano structure, such as the case of the structural displacement Δy in Supplementary Figures 5a - 5c, or localized dielectric perturbation $\Delta\epsilon$ in Supplementary Figures 5g - 5i.

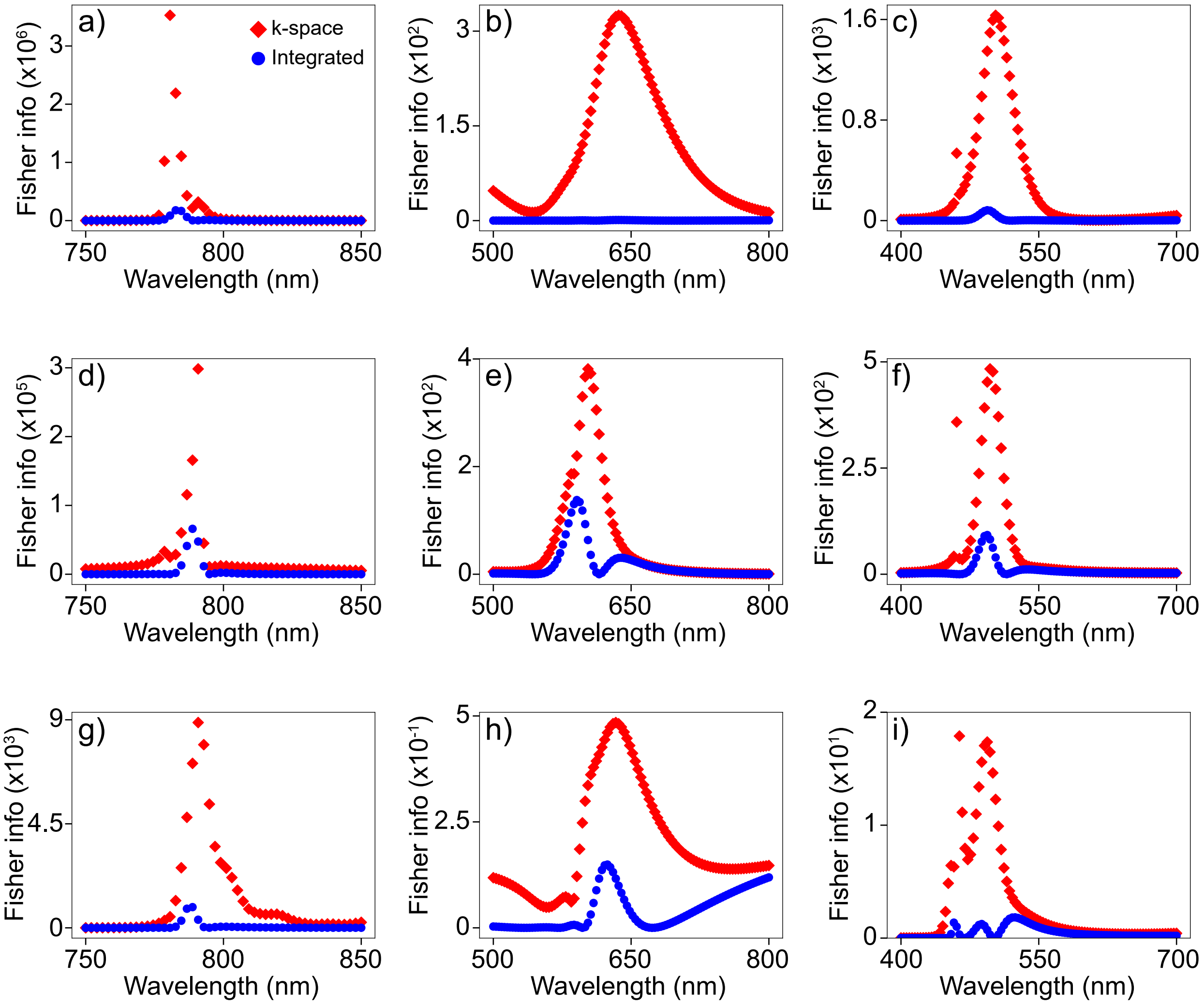

Supplementary Fig. 5: **Fisher information comparison for different Fano structures and perturbations.** Fisher information for an angle resolved Fourier space calculation and an angle integrated spectral calculation, indicated by the red diamonds and blue circles respectively. The panel labels correspond to calculations on geometries that correspond to the labels defined in Figure 3.

These asymmetries in the Fano structure induce asymmetries in Fourier space as well, ultimately leading to effective directional scattering events. Note that in the case of a symmetric perturbation, such as the homogeneous refractive index change in Supplementary Figures 5d-5f, there is still information to be gained from a Fourier space readout. This can be explained by slight differences in modal radiation patterns of the bright and dark modes, which generate symmetric residues in the acquired Fourier space modal interferograms.

References

(1) Association, E. M. V.; others Standard for characterization of image sensors and cameras. *EMVA Standard* **2010**, *1288*.
